# Association of sugar-sweetened beverage consumption and sleep quality with anxiety symptoms: a cross-sectional study of Tibetan college students at high altitude

**DOI:** 10.3389/fpsyg.2024.1383042

**Published:** 2024-03-27

**Authors:** Qin Qiu, Guangxin Chai, Shiming Xie, Tianyu Wu

**Affiliations:** School of Physical Education and Health, Jiangxi Science and Technology Normal University, Nanchang, Jiangxi, China

**Keywords:** high altitude, adolescents, eating behavior, lifestyle, anxiety symptoms

## Abstract

**Background:**

Research on the association between sugar-sweetened beverages (SSBs) consumption and sleep quality with anxiety symptoms has been highly emphasized. However, past studies have focused on college students in plains areas, while fewer research studies have been conducted on Tibetan college students at high altitudes. Whether this association changes due to ethnicity or altitude is unclear. The present study will contribute to the prevention and intervention of depressive symptoms among Tibetan college students at high altitude.

**Methods:**

A self-assessment questionnaire was administered to 3,026 university students (1,491 boys students, 49.27%) on SSBs consumption, sleep quality and anxiety symptoms status in the Tibetan Plateau, a high-altitude region of China. Logistic regression analysis and ordered logistic regression analysis in generalized linear model were used to analyze the association between SSBs consumption and sleep quality with anxiety symptoms.

**Results:**

The prevalence of anxiety symptoms among Tibetan college students at high altitude was 26.9%. SSBs consumption of ≤1 times/week, 2–5 times/week, and ≥ 6 times/week were 20.7, 28.1, and 45.7%, respectively, with statistically significant differences (*χ*^2^ value of 134.353, *p* < 0.001). Anxiety detection rates for Sleep quality of Good (PSQI ≤5), Moderate (PSQI 6–7), and Poor (PSQI >7) were 16.8, 19.8, and 32.0%, respectively, and the difference was also statistically significant (*χ*^2^ value was 73.761, *p* < 0.001). The ordered logistic regression analysis in the generalized linear model showed that, overall, the group of college students with SSBs ≤1 times/week and sleep quality of Good served as the reference group, and the group with SSBs ≥6 times/week and sleep quality of Poor (OR: 5.06, 95% CI: 3.75–6.83) had the highest risk of anxiety symptoms.

**Conclusion:**

SSBs consumption and sleep quality were associated with anxiety symptoms, and there was an interaction effect. Effective control of SSBs consumption and improvement of sleep quality may be important factors in preventing and reducing the occurrence of anxiety symptoms.

## 1 Introduction

As a common emotional and psychological disorder, anxiety symptoms are common among college students. Especially in the process of college students’ life and study, when they encounter some tension, stress, pressure, or even frustration, it is very easy to appear uneasy and worried psychological state. With the constant changes in modern lifestyles, the decreasing level of physical activity and prolonged screen time among college students, coupled with the increasing academic pressure on college students, the proportion of college students experiencing anxiety symptoms has been rising. Some studies have reported that 23.7% of college students in China suffer from anxiety symptoms of different degrees (Cheng et al., 2017). It has also been shown that the prevalence of anxiety symptoms among university medical students was 25.9%, and it was concluded that less social support (OR = 1.4) was an independent risk factor for the occurrence of anxiety symptoms (Liu et al., 2022a). A survey of the U.S. population showed that during the COVID-19 pandemic, the prevalence of anxiety symptoms among adults in the U.S. increased from 36.4 to 41.5%, with the greatest increase in adults aged 18–29 years, and the study suggests that the prevalence of anxiety symptoms among adults in this age group should be emphasized (Vahratian et al., 2021). If anxiety symptoms are not timely intervened and guided, they are very likely to develop into serious psychological problems and even suicidal behaviors, which will have extremely negative impacts on college students’ academic and future achievements (Ma et al., 2020; Wu et al., 2021; Zhang et al., 2022). Therefore, paying attention to college students anxiety symptoms is of great significance in promoting the healthy development of college students.

Several studies have found strong associations between anxiety symptoms and personality traits, life circumstances, social adjustment, age, gender, eating behaviors, sleep quality, etc. (Beesdo et al., 2009; Hoge et al., 2017; Peres et al., 2017; Meinlschmidt et al., 2022). Among the factors affecting anxiety symptoms, past studies have focused more on the association between sleep quality with anxiety symptoms, while fewer studies have examined the association between SSBs consumption and anxiety symptoms (Chellappa and Aeschbach, 2022). It is noteworthy that the negative health effects of excessive consumption of SSBs by college students have been emphasized in recent years (Audain et al., 2019). A meta-analysis showed an increased risk of the metabolic syndrome (MetS) in the highest group (OR: 1.18, 95% CI: 1.06, 1.32) compared with the group with the lowest SSBs consumption (Munoz-Cabrejas et al., 2023). Additionally, studies have confirmed that overconsumption of SSBs also leads to an increased risk of obesity and psychological problems (Malik and Hu, 2022). A U.S. survey showed that between 2011 and 2014, U. S. adults consumed 145 kcal per day from SSBs, equivalent to 6.5 percent of daily calories (Welsh et al., 2011). Another study also showed that of the risk factors predicting the development of type 2 diabetes over the next 10 years, 8.7% (95% CI: 3.9–12.9%) of cases in the United States and 3.6% (95% CI 1.7–5.6%) in the United Kingdom were attributable to an overabundance of SSBs consumption, suggesting that high levels of SSBs consumption among adults may be associated with a significant number of new-onset chronic diseases (Imamura et al., 2015). Past studies have also shown that sleep quality problems are prevalent among college students and have a negative impact on their mental health (Brand et al., 2019). A study confirmed a moderately significant effect of improved sleep on comprehensive mental health (*g* + = −0.53), as well as significant improvements in depression (*g* + = −0.63), anxiety (*g* + = −0.51), and rumination (*g* + = −0.49), and found that there was a dose–response relationship between sleep and mental health, whereby improvements in sleep quality lead to Improvement in mental health (Scott et al., 2021). Another study of 16–25 year olds also confirmed a significant association between poor sleep quality and negative mental health (Alonzo et al., 2021). It is clear that SSBs consumption and sleep quality have a negative impact on the mental health of college students. However, there is limited research on the association between SSBs consumption and sleep quality with anxiety symptoms among college students.

China’s Qinghai-Tibetan Plateau region is a typical high-altitude area in the world, and this area is mainly inhabited by Tibetans (Wu, 2001). High altitude areas are characterized by high radiation levels, low oxygen levels and low vegetation cover, which adversely affects physical fitness and mental health (Yi et al., 2010). In addition, Tibetans have lived at high altitudes for a long time, forming unique lifestyles and eating behaviors that differ greatly from those of groups in plains areas (Wei et al., 2017). Previous studies on anxiety symptoms among Tibetan college students at high altitude are very limited. In addition, no studies have been conducted on the association between SSBs consumption, sleep quality with anxiety symptoms among Tibetan college students at high altitude. Therefore, the present study was conducted to investigate the current status of SSBs consumption, sleep quality, and anxiety symptoms among 3,026 college students in Xining City, Qinghai Province, and Lhasa City, Tibet, in the Tibetan Plateau region of China, and to further analyze the associations among them. This study will provide necessary reference and assistance for mental health promotion and intervention for Tibetan college students in high altitude areas.

## 2 Methods

### 2.1 Participants

The selection of participants for this study was divided into the following steps. In the first step, Xining City, Qinghai Province (altitude 3,137 meters) and Lhasa City, Tibet (altitude 3,650 meters) in the Tibetan Plateau region of China were used as the regions for the selection of participants in this study. In the second step, two universities were randomly selected in each region as the schools sampled for this study. In the third step, among all classes from the first year of university to the fourth year of university in each university, 5 teaching classes with a high concentration of Tibetan students were randomly selected in each grade using random coded sampling. Tibetan college students who met the inclusion criteria for this study were included as participants in the self-assessment questionnaire.

**Figure 1:**
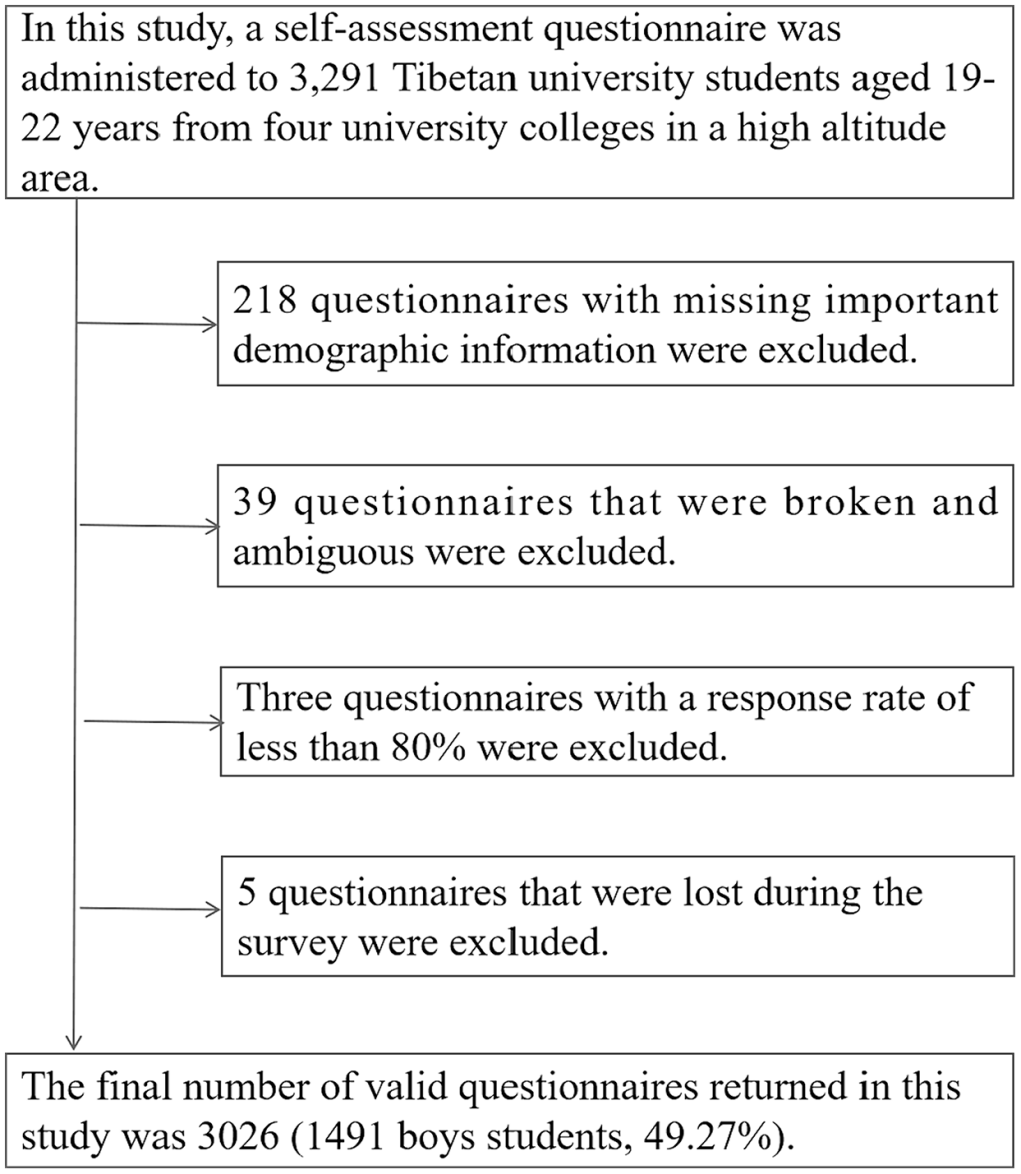
Sampling procedure for participants of Tibetan college students in high altitude areas of China.

The inclusion criteria for the participants in this study were: both the father and the mother were Tibetan, they had lived in Qinghai or Tibet for more than 3 years, the participants themselves did not have serious physical or mental illnesses, and the participants gave their informed consent and voluntarily accepted the survey of this study. Eventually, a total of 3,291 Tibetan college students aged 19–22 years old enrolled in 80 teaching classes at four universities were included in this study. At the end of the survey, 265 questionnaires with missing key demographic information, broken questionnaires, or ambiguous questionnaires were excluded, and finally 3,026 valid questionnaires were returned (1,491 boys students, 49.27%). The sampling procedure is shown in Figure 1, and written informed consent was obtained from the participants before the survey. This study was approved by the Ethics Committee of Jiangxi Science and Technology Normal University (IRB-JXSTNU-2022003).

### 2.2 Anxiety symptoms

Anxiety symptoms were investigated using the Self-rating Anxiety Scale (SAS) (Zung, 1971). The SAS consists of 20 entries, each of which consists of 4 choices: “No or little time,” “A little time,” “Quite a lot of time,” “Most or all of the time,” and “Most or all of the time.” Most or all of the time,” each of which is scored from 1 to 4. Participants filled out the questionnaire according to their actual situation in the past 7 days. Questions 5, 9, 13, 17, and 19 were reverse scored. The total score of the questionnaire was obtained by summing the 20 entries, and the total score of the questionnaire was multiplied by 1.25 to obtain the standardized score, which ranged from 25 to 100. Higher participant scores indicated more severe anxiety symptoms. SAS standardized scores ≥50 were determined to be the presence of anxiety symptoms in the participants (Zung, 1974). The SAS is widely used in the Chinese adult population and has good reliability and validity, the Cronbach’s alpha was 0.87 (Wang and Zhao, 2020; Kim et al., 2022; Hao and Zhang, 2023). The Cronbach’s alpha was 0.86 in the present study.

### 2.3 Sugar-sweetened beverage (SSBs)

Sugar-sweetened beverages (SSBs) consumption were self-assessed using the Beverage Intake Questionnaire (BEVQ15) (Hedrick et al., 2012; Fausnacht et al., 2020). The questionnaire was self-assessed to investigate participants’ consumption of the 15 beverages included over the past month. This included the frequency of consumption and how many milliliters were consumed per drink. The types of beverages included fruit juices, sweetened cocktail juices, chocolate milk, black coffee, sweetened nut juices, carbonated drinks, energy drinks, and sweetened milk tea. The questionnaire was designed with the frequency of SSBs categorized as <1 time/week, 1 time/week, 2–3 times/week, 4–6 times/week, 1 time/day, 2 times/day, and > 3 times/day. Participants made a single choice based on their own actual situation in the past 30 days. The amount of SSBs was calculated using milliliters, each time based on a can of Coke 330 mL. In this study, SSBs were categorized as ≤1 times/week, 2–5 times/week, and ≥ 6 times/week with reference to the categorization method of previous studies.

### 2.4 Sleep quality

In this study, the pittsburgh sleep quality index (PSQI) was used to conduct a self-assessment questionnaire for sleep quality (Sampol et al., 2023). The PSQI questionnaire is widely used in countries around the world and is the most commonly used self-assessment scale for evaluating sleep quality. The PSQI has good reliability and validity in evaluating the sleep quality of Chinese people (Chang et al., 2021; Liu et al., 2022b; Sun et al., 2023). The PSQI questionnaire consists of 18 items divided into 7 dimensions, namely, sleep efficiency, duration of sleep at night, daytime dysfunction, use of hypnotic medication, subjective sleep quality, sleep disorders, and sleep latency. Participants filled in the questionnaire and selected the entries according to their actual situation in the past 30 days. The total score of the PSQI questionnaire was 21, and the higher the score of the participant, the worse the sleep quality. Based on the participants’ scores, sleep quality was categorized as good, moderate, and poor, with ≤5 points, 6–7 points, and > 7 points, respectively.

### 2.5 Covariates

Covariates in this study included Parents’ educational level, Socio economic status (SES), Body mass index (BMI), Screen Time and Frequency of study breakfast, MVPA.

(1) Parents’ educational level was based on the highest educational level of either the father or the mother, as indicated by the participants. In this study, Parents’ educational level was categorized as Primary and below, Junior high school, College or above.
(2) Socioeconomic status (SES) survey was evaluated using the International Socio-Economic Index (ISEI). This index reflects the socio-economic status of people in terms of education, income and occupation. The index was developed by Ganzeboom, Graaf and Treiman in the 1990s (Ganzeboom et al., 1992). Following the standardized calculation procedure provided by Ganzeboom and Treiman (1996), we used SPSS 25.0 to convert participant-completed occupation codes to ISEI. ISEI is a continuous variable, with larger values indicating higher SES. In this study, the scores were categorized as Low (<15th), Medium (15-85th), and High (>85th).
(3) Body mass index (BMI) was calculated based on participants’ height and weight. It is weight (Kg)/height (m)^2^. Based on the calculation results, it was categorized as Underweight for ≦18.4Kg/m^2^, Normal for 18.5–23.9Kg/m^2^, Overweight for 24.0–27.9Kg/m^2^, and Obese for ≧28Kg/m^2^ according to the classification criteria. The height and weight tests were conducted according to the testing methods and instruments required by the Physical Fitness Standard for Chinese Students. The results of the height test were accurate to 0.1 centimeter. The results of the weight test are accurate to 0.1 kg.
(4) The Screen Time test mainly investigated the average length of time participants watched TV, cell phones, and tablets in the past 7 days. According to the relevant standards, it was categorized into ≤120 min/d, > 120 min/d (McGough, 2021).
(5) Frequency of breakfast was investigated using a self-assessment questionnaire. The corresponding entries were selected based on the participants’ actual situation in the past 7 days. In this study, it was categorized as ≤1 times/week, 2–5 times/week, and ≥ 6 times/week.
(6) The Moderate and Vigorous Physical Activity (MVPA) survey was conducted using the entries of the physical activity section of the China National Student Physical Health Research Questionnaire. The participants were surveyed on the average frequency and duration of MVPA participation per day over the past 7 days, which was used to calculate the length of MVPA per day for the subjects. Specific items of MVPA included ball sports, skiing, fast running, and bicycling. In this study MVPA was categorized as <30 min/d, 30–60 min/d, and > 60 min/d according to the participants’ average time in the past 7 days.

### 2.6 Quality control

The survey of this study was conducted by self-assessment of the questionnaire. The questionnaire survey was conducted by teachers who were trained and qualified by the subject team as survey staff. Divided into four groups of two people each, they entered each school to conduct the questionnaire survey on site. The purpose and requirements of the survey were explained to the participants before the questionnaire survey. Participants were also asked to sign an informed consent form before the survey. The questionnaires were distributed, filled out on the spot and returned on the spot. Each questionnaire took about 15–20 min to complete. When the questionnaires were returned, the survey staff checked the completeness of the questionnaires completed by the participants. Participants were asked to fill in any missing or incorrect information. The height and weight tests were conducted by a person who was responsible for calibrating the instruments before each day’s test in order to ensure the accuracy of the tests.

### 2.7 Statistical analysis

The anxiety symptoms of Tibetan college students at high altitude were characterized by percentages. Comparison of the detection rates of anxiety symptoms among different categories of college students was performed by means of the chi-square test. The relationship between Sugar-sweetened beverage and Sleep quality with anxiety symptoms in Tibetan college students at high altitude was analyzed by hierarchical logistic regression analysis. Participants were analyzed with the presence of anxiety symptoms as the dependent variable, and different sugar-sweetened beverage and sleep quality as independent variables. Model 1 was not adjusted for any covariates, and Model 2 was adjusted for age, parental education level, SES, and BMI based on Model 1. Model 3 adjusted for screen time, frequency of breakfast, and MVPA on the basis of Model 2. The analyses of the relationship between the interaction effects of Sugar-sweetened beverage and Sleep quality with anxiety symptoms were analyzed using the method of Ordered Logistic Regression Analysis in Generalized Linear Models. Ordered Logistic Regression adjusted for age, parental education, SES, BMI, screen time, breakfast frequency, and MVPA. The results of the analyses were reported odds ratios (OR) and 95% confidence interval (CI), respectively.

Data processing and analysis were performed using SPSS 25.0 (SPSS Inc., Chicago, IL, USA). A two-sided test level of *α* = 0.05 was used.

## 3 Results

In this study, a self-assessment questionnaire on sugar-sweetened beverage, sleep quality and anxiety symptoms was administered to 3,026 Tibetan college students aged 19–22 years at high altitude. The prevalence of anxiety symptoms among Tibetan college students at high altitude was 26.9% (813/3026). The prevalence of anxiety symptoms among boys was 26.0% (387/1491) and among girls was 27.8% (426/1535), and the difference was not statistically significant (*χ*^2^ value of 1.243, *p* = 0.265). The detection rates of anxiety with SSBs of ≤1 times/week, 2–5 times/week, and ≥ 6 times/week were 20.7, 28.1, and 45.7%, respectively, and the difference was statistically significant (*χ*^2^ value of 134.353, *p* < 0.001). The detection rates of anxiety in sleep quality of Good (PSQI ≤5), Moderate (PSQI 6–7), and Poor (PSQI >7) were 16.8, 19.8, and 32.0%, respectively, and the difference was also statistically significant (*χ*^2^ value was 73.761, *p* < 0.001).

Overall, the prevalence of anxiety symptoms was compared in terms of SES, BMI, screen time, and frequency of breakfast for different covariates, and the differences were statistically significant (*χ*^2^ values of 12.085, 16.316, 19.664, and 96.473, *p* < 0.01). Comparison of the detection rates of anxiety symptoms for other covariates in terms of different genders is shown in Table 1.

**Table 1:** Comparison of the detection rate of anxiety symptoms among different categories of college students in Tibetan at high altitude (%).

Classifications	Boys (*n* = 1,491)			Girls (*n* = 1,535)			Total (*n* = 3,026)		
	*N* (%)	Chi-Square	*p*-value	*N* (%)	Chi-Square	*p*-value	*N* (%)	Chi-Square	*p*-value
Age (years)
19	109 (24. 4)	10.492	0.015	159 (30.3)	6.762	0.08	268 (27.6)	1.709	0.635
20	103 (23.6)			132 (27.1)			235 (25.4)		
21	89 (25.1)			99 (28.6)			188 (26.9)		
22	86 (34.0)			36 (20.3)			122 (28.4)		
Parents’ educational level
Primary and below	89 (30.1)	3.381	0.184	64 (25.7)	0.913	0.634	153 (28.1)	0.532	0.766
Junior high school	269 (25.1)			317 (27.9)			586 (26.5)		
College or above	29 (23.6)			45 (30.0)			74 (27.1)		
Socioeconomic status (SES)
Low (<15th)	82 (32.3)	6.430	0.040	75 (34.6)	6.027	0.049	157 (33.3)	12.085	0.002
Medium (15-85th)	256 (24.8)			295 (26.9)			551 (25.9)		
High (>85th)	49 (24.0)			56 (25.5)			105 (24.8)		
Body mass index (BMI)
Underweight	49 (30.8)	3.333	0.343	67 (19.8)	41.432	<0.001	116 (23.3)	16.316	0.001
Normal	197 (25.0)			212 (25.8)			409 (25.4)		
Overweight	82 (27.6)			41 (33.1)			123 (29.2)		
Obese	59 (23.8)			106 (42.6)			165 (33.2)		
Screen Time
≤ 120 min/d	84 (19.4)	13.648	<0.001	86 (22.8)	6.256	0.012	170 (21.0)	19.664	<0.001
>120 min/d	303 (28.6)			340 (29.4)			643 (29.0)		
Frequency of breakfast
≤1 times/week	125 (47.7)	91.926	<0.001	55 (42.3)	18.985	<0.001	180 (45.9)	96.473	<0.001
2–5 times/week	126 (27.3)			113 (30.4)			239 (28.7)		
≥6 times/week	136 (17.7)			258 (25)			394 (21.9)		
MVPA
<30 min/d	262 (26.4)	0.446	0.800	374 (28.7)	6.091	0.048	636 (27.7)	3.967	0.138
30–60 min/d	96 (25.3)			43 (20.8)			139 (23.7)		
>60 min/d	29 (24.0)			9 (33.3)			38 (25.7)		

The results of this study showed that in terms of gender, the prevalence of anxiety symptoms in different SSBs consumption college boys and girls were statistically significant (*χ*^2^ values of 109.881, 36.358, *p* < 0.001). In terms of sleep quality, the prevalence of anxiety symptoms in college students boys and girls were also statistically significant (*χ*^2^ values were 52.211, 24.444, *p* < 0.001). The specific results are shown in Table 2.

**Table 2:** Univariate analysis of different SSBs consumption and sleep quality with anxiety symptoms in Tibetan college students at high altitude (%).

Classifications	Anxiety symptoms (SAS ≥ 50)											
	Boys (*n* = 1,491)				Girls (*n* = 1,535)				Total (*n* = 3,026)			
	Yes	No	Chi-Square	*p*-value	Yes	No	Chi-Square	*p*-value	Yes	No	Chi-Square	*p*-value
	*N* (%)	*N* (%)			*N* (%)	*N* (%)			*N* (%)	*N* (%)		
Sugar-sweetened beverages (SSBs)
≤1 times/week	160 (18.3)	714 (81.7)	109.881	<0.001	218 (23.0)	730 (77.0)	36.358	<0.001	378 (20.7)	1,444 (79.3)	134.353	<0.001
2–5 times/week	70 (24.1)	220 (75.9)			113 (31.2)	249 (68.8)			183 (28.1)	469 (71.9)		
≥6 times/week	157 (48.0)	170 (52.0)			95 (42.2)	130 (57.8)			252 (45.7)	300 (54.3)		
Sleep quality
Good (PSQI ≤5)	71 (16.1)	371 (83.9)	52.211	<0.001	60 (17.9)	276 (82.1)	24.444	<0.001	131 (16.8)	647 (83.2)	73.761	<0.001
Moderate (PSQI 6–7)	25 (15.7)	134 (84.3)			36 (24.2)	113 (75.8)			61 (19.8)	247 (80.2)		
Poor (PSQI >7)	291 (32.7)	599 (67.3)			330 (31.4)	720 (68.6)			621 (32.0)	1,319 (68.0)		

MVPA, moderate-to-vigorous physical activity; PSQI, Pittsburgh Sleep Quality Index.

**Figure 2:**
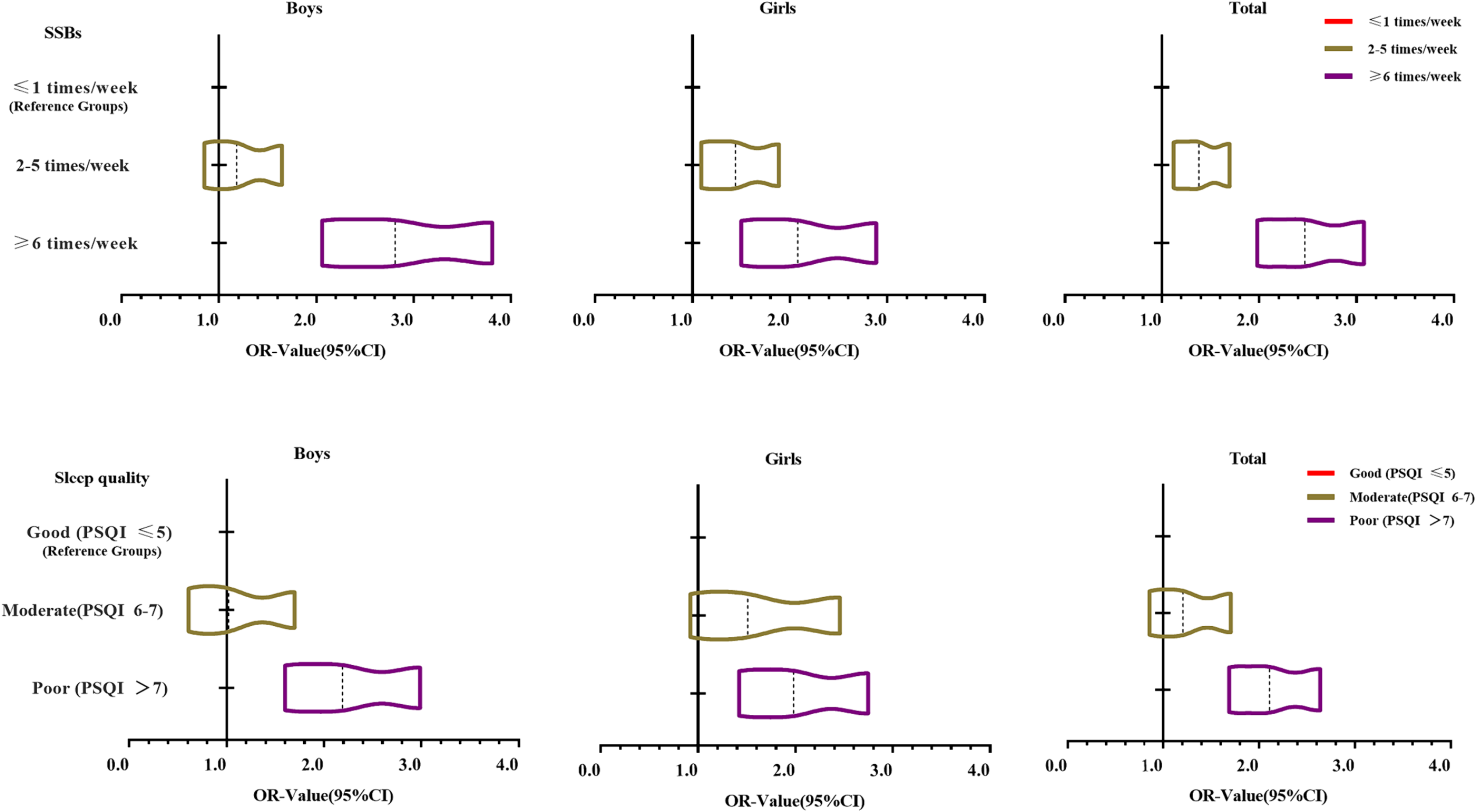
Logistic regression OR values of SSBs consumption and sleep quality with anxiety symptoms of Tibetan college students in high-altitude areas.

The anxiety symptoms of college students at high altitude were used as dependent variables. The independent variables were sugar-sweetened beverages and sleep quality. Logistic regression analysis was performed after stratifying by gender. For logistic regression analysis, Model 1 was not adjusted for any covariates, Model 2 was adjusted for age, parental level of education, SES, and BMI on the basis of Model 1, and Model 3 was adjusted for screen time, frequency of breakfast, and MVPA on the basis of Model 2. Overall, the results of Model 3 showed that, using the group with SSBs ≤1 times/week as a reference, SSBs consumption of 2–5 times/week (OR: 1.38, 95% CI: 1.12–1.70) and SSBs consumption of ≥6 times/week (OR: 2.47, 95% CI: 1.98–3.08) of college students had a higher risk of anxiety symptoms (*p* < 0.01). Using the group with Sleep quality of Good (PSQI ≤5) as a reference, college students in the group with Sleep quality of Poor (OR: 2.11, 95% CI: 1.69 ~ 2.64) had a higher risk of developing anxiety symptoms (*p* < 0.001). The results of the study by gender are shown in Table 3. Trends in logistic regression ORs for SSBs consumption and sleep quality with anxiety symptoms are shown in Figure 2.

**Table 3:** Logistic regression analysis of SSBs consumption and sleep quality with anxiety symptoms of Tibetan college students in high-altitude areas.

Sex	Categorization		Model 1		Model 2		Model 3	
			OR (95% CI)	*p-*value	OR (95% CI)	*p-*value	OR (95% CI)	*p-*value
Boys	Sugar-sweetened beverages (SSBs)	≤1 times/week	1.00		1.00		1.00		2–5 times/week	1.42 (1.03 ~ 1.95)	0.031	1.36 (0.99 ~ 1.88)	0.058	1.18 (0.85 ~ 1.65)	0.314	≥6 times/week	4.12 (3.13 ~ 5.43)	<0.001	4.04 (3.06 ~ 5.34)	<0.001	2.81 (2.06 ~ 3.81)	<0.001	Sleep quality (PSQI)	Good	1.00		1.00		1.00		Moderate	0.98 (0.59 ~ 1.60)	0.920	1.00 (0.61 ~ 1.64)	0.985	1.02 (0.61 ~ 1.7)	0.941	Poor	2.54 (1.9 ~ 3.39)	<0.001	2.5 (1.87 ~ 3.34)	<0.001	2.19 (1.6 ~ 2.99)	<0.001
Girls		Sugar-sweetened beverages (SSBs)	≤1 times/week	1.00		1.00		1.00		2–5 times/week	1.52 (1.16 ~ 1.99)	0.002	1.50 (1.14 ~ 1.97)	0.004	1.44 (1.09 ~ 1.89)	0.010	≥6 times/week	2.45 (1.8 ~ 3.32)	<0.001	2.43 (1.78 ~ 3.32)	<0.001	2.08 (1.50 ~ 2.89)		<0.001	Sleep quality (PSQI)	Good	1.00		1.00		1.00		Moderate	1.47 (0.92 ~ 2.34)	0.109	1.48 (0.92 ~ 2.38)	0.107	1.51 (0.92 ~ 2.46)	0.102	Poor	2.11 (1.55 ~ 2.87)	<0.001	2.07 (1.51 ~ 2.83)	<0.001	1.98 (1.42 ~ 2.75)	<0.001
Total	Sugar-sweetened beverages (SSBs)		≤1 times/week	1.00		1.00		1.00		2–5 times/week	1.49 (1.21 ~ 1.83)	<0.001	1.48 (1.21 ~ 1.82)	<0.001	1.38 (1.12 ~ 1.70)	0.003	≥6 times/week	3.21 (2.62 ~ 3.93)	<0.001	3.18 (2.59 ~ 3.89)	<0.001	2.47 (1.98 ~ 3.08)	<0.001	Sleep quality (PSQI)		Good	1.00		1.00		1.00		Moderate	1.22 (0.87 ~ 1.71)	0.248	1.20 (0.86 ~ 1.68)	0.293	1.21 (0.86 ~ 1.71)	0.283	Poor	2.33 (1.88 ~ 2.87)	<0.001	2.30 (1.86 ~ 2.84)	<0.001	2.11 (1.69 ~ 2.64)	<0.001

Model 1 was not adjusted for any covariates, and Model 2 was adjusted for age, parental education level, SES, and BMI based on Model 1. Model 3 adjusted for screen time, frequency of breakfast, and MVPA on the basis of Model 2. Good (PSQI ≤ 5), Moderate (PSQI 6–7), Poor (PSQI >7).

The presence of anxiety symptoms among Tibetan college students at high altitude was used as the dependent variable. SSBs consumption and Sleep quality were used as independent variables. Ordered Logistic Regression in Generalized Linear Model was used to analyze the association between the interaction effects of SSBs consumption and Sleep quality with anxiety symptoms among Tibetan college students. Ordered Logistic Regression adjusted for age, parental level of education, SES, BMI, screen time, frequency of breakfast, and MVPA. Overall, the college students SSBs ≤1 times/week and sleep quality of Good group were used as the reference group, and the college students with SSBs ≥6 times/week and sleep quality of Poor group (OR: 5.06, 95% CI: 3.75–6.83) had the highest risk of anxiety symptoms. Students had the highest risk of developing anxiety symptoms. Significantly, in terms of gender, college students in the SSBs ≥6 times/week and sleep quality of Poor group had a higher risk of developing anxiety symptoms among boys (OR: 7.61, 95% CI: 4.94–11.71) than girls (OR: 3.26, 95% CI 2.13 ~ 5.00) (*p* < 0.001).

Other specific results are shown in Table 4.

**Table 4:** Order regression analysis of interaction effects of SSBs consumption and sleep quality with anxiety symptoms in Tibetan college students at high altitude.

Sex	Classification of interaction		Ordered Logistic Regression	
	Sugar-sweetened beverages (SSBs)	Sleep quality (PSQI)	OR (95% CI)	*p*-value
Boys	≤1 times/week	Good	1.00		Moderate	0.99 (0.48 ~ 2.04)	0.983	Poor	2.62 (1.73 ~ 3.97)	<0.001	2–5 times/week	Good	1.36 (0.68 ~ 2.70)	0.386	Moderate	1.94 (0.79 ~ 4.76)	0.149	Poor	3.52 (2.16 ~ 5.73)	<0.001	≥6 times/week	Good	9.18 (4.62 ~ 18.22)	<0.001	Moderate	3.51 (1.35 ~ 9.15)	0.010	Poor	7.61 (4.94 ~ 11.71)	<0.001
Girls	≤1 times/week	Good	1.00		Moderate	1.16 (0.67 ~ 2.03)	0.595	Poor	1.29 (0.89 ~ 1.87)	0.176	2–5 times/week	Good	0.35 (0.14 ~ 0.85)	0.021	Moderate	1.68 (0.75 ~ 3.74)	0.208	Poor	2.50 (1.66 ~ 3.76)	<0.001	≥6 times/week	Good	1.56 (0.62 ~ 3.96)	0.346	Moderate	1.34 (0.26 ~ 6.85)	0.725	Poor	3.26 (2.13 ~ 5.00)	<0.001
Total	≤1 times/week	Good	1.00		Moderate	1.16 (0.75 ~ 1.79)	0.501	Poor	1.85 (1.40 ~ 2.43)	<0.001	2–5 times/week	Good	0.74 (0.44 ~ 1.27)	0.273	Moderate	1.83 (1.01 ~ 3.32)	0.047	Poor	3.11 (2.28 ~ 4.23)	<0.001	≥6 times/week	Good	4.53 (2.68 ~ 7.68)	<0.001	Moderate	2.33 (1.04 ~ 5.25)	0.041	Poor	5.06 (3.75 ~ 6.83)	<0.001

Ordered Logistic Regression adjusted for age, parental level of education, SES, BMI, screen time, frequency of breakfast, and MVPA. Good (PSQI ≤ 5), Moderate (PSQI 6–7), Poor (PSQI > 7).

## 4 Discussions

High-altitude areas have a special natural and human environment. The scarcity of oxygen and the long hours of sunshine in this region pose many challenges to human survival (Wang et al., 2018). The Tibetan Plateau region of China is a typical high-altitude area in the world, and this region is mainly dominated by Tibetans, who have long formed their own special lifestyle and dietary behaviors. However, fewer studies have been conducted on the anxiety symptoms of Tibetan college students in this region. The results of this study show that the prevalence of anxiety symptoms among Tibetan college students in high-altitude areas of China is 26.9%, and there is no gender difference in the detection rate of anxiety between boys and girls. Some scholars showed that the prevalence of anxiety symptoms among Chinese college students was 31.0%, which was higher than the results of the present study (Xiang et al., 2020). A survey of college students in Bangladesh confirms that 82.5% of college students have mild anxiety symptoms and 14.08% have extreme anxiety, and that factors such as gender, family size, and area of residence are important factors affecting anxiety levels. Gender, family size, and living area are all important factors affecting the anxiety level of students (Hoque et al., 2021). A survey of Pakistani medical students also confirmed the presence of anxiety symptoms in 3.4% of college students, with sex and age differences (Junaid et al., 2022). It is evident that the findings of anxiety symptoms among college students vary considerably from region to region. However, the results of this study are sufficient to show that the prevalence of anxiety symptoms among Tibetan college students in high altitude areas of China should be emphasized and paid attention to. The study confirms that the occurrence of anxiety symptoms, if not promptly paid attention to, will lead to serious mental illnesses and even suicidal behaviors, which will have a negative impact on the health of college students (Li et al., 2022). There are also studies confirming that the prevalence of anxiety symptoms is rising rapidly with the increasing pressure of life and schooling among adolescents, and calling for attention to be paid to them (Andrews and Wilding, 2004). The results of this study show that the occurrence of anxiety symptoms does not change according to the altitude of the altitude. It is important to pay attention to the anxiety symptoms of Tibetan college students at high altitude. Therefore, it is especially important to analyze the causes of anxiety symptoms among Tibetan college students at high altitude.

A number of studies in the past have confirmed that the causes of anxiety symptoms include dietary behaviors, lifestyle habits, and many other aspects (Regehr et al., 2013; O'Rourke et al., 2020). Among these many factors affecting anxiety symptoms, it is particularly important to study them in the context of poor dietary and habitual problems prevalent among current college students. SSBs consumption are the most common problem among adolescents today. Studies have confirmed that excessive SSBs can lead to chronic cardiovascular disease and obesity, which can have a negative impact on physical health, as well as mental health, which can be extremely detrimental (Park et al., 2022). In addition, sleep quality should not be overlooked among the factors that negatively affect adolescent health. Research has confirmed that sleep quality has a negative impact on both physical and mental health (Leow et al., 2023). Adolescents with poorer sleep quality are at higher risk for obesity, mental health problems (Zhang et al., 2018). However, findings on SSBs consumption and sleep quality with mental health are not entirely consistent. Studies have confirmed that the relationship between SSBs consumption and health status varies somewhat with ethnicity, region of life, age, and gender (Otto et al., 2022). The relationship between sleep quality and mental health also changes considerably depending on the study population, geographic area, and age. Therefore, it is particularly important for this study to investigate the relationship between SSBs consumption and sleep quality with anxiety symptoms in Tibetan college students at high altitude.

This study analyzed anxiety symptoms affecting Tibetan college students at high altitude from the perspective of SSBs consumption and sleep quality, which are the most common in current adolescents. The results of this study confirmed that college students with SSBs consumption of 2–5 times/week and SSBs of ≥6 times/week had a higher risk of anxiety symptoms compared to the group of Tibetan college students with SSBs ≤1 times/week at high altitude. This result did not change according to gender. This suggests that there is an association between SSBs consumption and anxiety symptoms among Tibetan college students at high altitude. The study confirms that excessive SSBs consumption are closely associated with an increased risk of anxiety symptoms and may be an important risk factor for the development of anxiety symptoms (von Philipsborn et al., 2019). In terms of sleep quality, the results of this study showed that the risk of anxiety symptoms was significantly increased in Tibetan college students in the group with sleep quality of Poor, using the group with sleep quality of Good as a reference. This result suggests that there is a strong association between sleep quality and anxiety symptoms among Tibetan college students at high altitude. The study confirms that a decrease in sleep quality will lead to hormone secretion disorders in the body, which will cause greater emotional fluctuations, and coupled with the influence of academic stress, it will be very easy to develop anxiety symptoms (Zisapel, 2018). The analysis of anxiety symptoms by the interaction effect of SSBs consumption and sleep quality in the present study also showed that the risk of anxiety symptoms among college students generally tended to increase with the increase of SSBs consumption and the decrease of sleep quality. The results of this study confirm that there is a close association between SSBs consumption and sleep quality with anxiety symptoms in Tibetan college students at high altitude, and that there is also a close association between the interaction effects of SSBs consumption and sleep quality on anxiety symptoms. Thus, excessive SSBs consumption and poor sleep quality may be important risk factors for the occurrence of anxiety symptoms in Tibetan college students at high altitude. The results of this study provide some reference for the prevention and intervention of anxiety symptoms in Tibetan college students at high altitude.

There are certain strengths and limitations of this study. Strengths: To the best of our knowledge, this study is the first to analyze the association relationship between SSBs consumption and sleep quality with anxiety symptoms among Tibetan college students in high-altitude areas in China, which will have a positive effect on the future development of mental health of Tibetan college students in high-altitude areas. In addition, this study investigates and analyzes Qinghai and Tibet, which are typical high-altitude areas on the Tibetan Plateau, with distinctive regional and ethnic characteristics, and contributes positively to enriching the research results in this field. However, this study also has some limitations. First, this study used a self-assessment survey to analyze SSBs consumption and sleep quality with anxiety symptoms, and there may be some bias between its results and the real situation. In the future, it is necessary to use precise instruments, such as accelerometers, to assess sleep quality. Secondly, this study is a cross-sectional survey study, which can only analyze the existence of correlations, but not the existence of causal associations. Cohort studies should be conducted in the future to analyze the causal relationship.

## 5 Conclusion

The results of this study show that there is an association between SSBs consumption and sleep quality with anxiety symptoms in Tibetan college students at high altitude in China. SSBs consumption and sleep quality may be important factors affecting anxiety symptoms in Tibetan college students at high altitude. Controlling SSBs consumption and improving sleep quality may be an effective way to reduce the occurrence of anxiety symptoms in Tibetan college students at high altitude. The results of this study may provide necessary reference and help for future prevention and intervention of college students anxiety symptoms in high altitude areas.

## Data availability statement

The raw data supporting the conclusions of this article will be made available by the authors, without undue reservation.

## Ethics statement

The studies involving humans were approved by this study was approved by the Ethics Committee of Jiangxi Science and Technology Normal University (IRB-JXSTNU-2022003). The studies were conducted in accordance with the local legislation and institutional requirements. The participants provided their written informed consent to participate in this study.

## Author contributions

QQ: Conceptualization, Investigation, Methodology, Project administration, Resources, Software, Supervision, Writing – original draft, Writing – review & editing. GC: Conceptualization, Funding acquisition, Investigation, Methodology, Validation, Visualization, Writing – original draft, Writing – review & editing. SX: Data curation, Formal analysis, Writing – original draft, Writing – review & editing. TW: Data curation, Formal analysis, Writing – original draft, Writing – review & editing.
